# Influence of IL10 (G1082A) and TNF**α** (G308A) Polymorphisms on the Survival of Pediatric Patients with ALL

**DOI:** 10.1155/2012/692348

**Published:** 2012-10-02

**Authors:** Dayse Maria Vasconcelos de Deus, Karina Araújo Lugo, Maria Tereza Cartaxo Muniz

**Affiliations:** ^1^Biological Sciences Institute, Pediatric Hematology Oncology Center (CEONHPE/UPE), Avenida Agamenon Magalhães, Bairro de Santo Amaro, 50100-010 Recife, PE, Brazil; ^2^Biological Sciences Institute, University of Pernambuco (UPE), Avenida Agamenon Magalhães, Bairro de Santo Amaro, 50100-010 Recife, PE, Brazil

## Abstract

Interleukin 10 (IL10) is a pleiotropic cytokine that stimulates various hematopoietic cells. The tumor necrosis factor alpha (TNF**α**) is a cytokine that may influence the transcriptional activity induced by glucocorticoids. This study examined the impact of TNF**α** (G308A) and IL10 (G1082A) polymorphisms at promoter regions in relation to the overall survival of 105 children (0 ≤ 18 years) with acute lymphoblastic leukemia (ALL) for a period of 126 months, treated according to the protocol GBTLI99. The G1082A and G308A polymorphisms were identified by allele-specific PCR and PCR-RFLP, respectively. Patients with IL10AA genotype had a higher death ratio (44%, *P* = 0.0089). Patients with both IL10AA and TNFAA genotypes showed the worst survival when compared with the IL10GG and TNFGA genotypes (*P* = 0.0043). The results of this study revealed a lower survival among patients with IL10AA genotype and the concomitant occurrence of IL10AA and TNFAA genotypes.

## 1. Introduction

 Glucocorticoids were among the first drug classes used in the treatment of patients with ALL and are still essential components of treatment [1]. The glucocorticoid receptors (GR) can form dimers, translocate to the nucleus, and interact with glucocorticoid-response elements to transactivate gene expression, or they can remain as monomers and repress the activity of transcription factors such as the activating protein-1 (AP-1) and nuclear factor-*κ*B (NF*κ*B) [2].

 Traditionally, prednisone has been the glucocorticoid's most commonly used drug in the treatment of patients with acute lymphoblastic leukemia (ALL). It is typically given for 4 consecutive weeks in combination with vincristine, anthracycline, asparaginase, and intrathecal chemotherapy. In the past few years, dexamethasone, another glucocorticoid, has been increasingly used to treat ALL. The biological background of prednisone response is still unknown. However, there have been investigations with respect to glucocorticoid receptors [3], distribution of GR isoforms [4], and genetic polymorphisms [5].

 Polymorphisms at the promoter region of IL10 gene are associated with several diseases, including autoimmune, infectious, cancer, Alzheimer's disease (AD), and lymphoblastic leukemia [6]. Subsequently, pleiotropic inhibitory and stimulatory effects on various types of blood cells were described for IL-10, including its role as a survival and differentiation factor for B cells. IL10 is produced by activated monocytes and T cells [7]. 

 A polymorphism at -1082 position, within the IL-10 gene promoter region, is known to have an influence on IL-10 plasma levels. The homozygous patients for the IL-10G allele showed high IL-10 plasma levels [8]. Franchimont et al. [9] have shown that the binding capacity for dexamethasone can be upregulated by high IL-10 production.

 Tumor necrosis factor (TNF) is a cytokine with pleiotropic biological activities, including the induction of programmed cell death (apoptosis) and the regulation of immune cell proliferation and differentiation [10], and may influence the transcriptional activity induced by the glucocorticoid of the glucocorticoid receptor gene [11].

 Although genetic polymorphisms in the TNF locus have been implicated in the severity of several B-cell lymphoproliferative diseases [12, 13], to date, only a few studies have found an association among the rare TNF AA of G308A polymorphism, the increased production of TNF protein, and the non-Hodgkin's lymphomas (NHL) susceptibility. 

 Currently, there are few studies related to the overall survival of patients with acute lymphoid leukemia and G1082A and G308A polymorphisms. In this study, the association of TNF*α* (G308A) and IL10 (G1082A) polymorphisms was analyzed in relation to the overall survival of Brazilian children with ALL, observed for a long time according to the GBTLI99 treatment protocol.

## 2. Materials and Methods

### 2.1. Patients and Samples

 The study group was composed of children with acute lymphoblastic leukemia, aged 0 ≤ 18 years, who were being evaluated at the Oncohematology Pediatric Center Oswaldo Cruz Hospital (HUOC), Recife, Brazil, since January 2004 to December 2011. We analyzed 105 patients for TNF*α* (G1082A) and IL10 (G308A) polymorphisms according to the overall survival, presenting clinical and laboratory diagnosis for acute lymphoblastic leukemia. Patient samples were collected from the bone marrow puncture in the posterior iliac crest. The DNA samples were obtained by salting out method [14]. The treatment was in accordance with the protocol of Brazilian group for the treatment of lymphoblastic leukemia in childhood (GBTLI99—protocol of Brazilian group for treatment of lymphoblastic leukemia in childhood) [15].

### 2.2. Genotyping

 The TNF*α* gene (G308A) was genotyped by PCR-RFLP method. The primers [13] used for genotyping were (forward) 5′-AGG CAA TAG GTT TTG AGG GC-3′ and (reverse) 5′-TCC TCC CTG CTC CGA TTC CG-3′. The cycling included 1 cycle of 95°C/6 min; 40 cycles of 95°C/60 s, 62.5°C/90 s, and 72°C/60 s; 1 cycle of 72°C/7 min. Thirty microliters of PCR mixture comprised 3.0 *μ*L Buffer (10x), 1.5 *μ*L MgCl_2_ (50 mM), 1 U Taq polymerase (5 U), 0.6 *μ*L dNTP (10 mM), and 0.6 *μ*L primer (10 pmol/*μ*L). The 308 G → A base pair substitution creates a *NcoI* restriction site. The PCR product (107 bp) of C308T was digested for 16 hours at 37°C using *NcoI* and analyzed on agarose gel 4% with ethidium bromide (0.4 mg/mL) in electrophoresis. The digestion of PCR product with *NcoI* showed fragments of 107 bp for AA genotype and 87 bp and 23 bp for the GG genotype.

 The IL10 gene polymorphism (G1082A) was genotyped by PCR allele-specific. The primers [16] used for genotyping were allele A forward-5′-CCT ATC CCT ACT TCC CCT-3′ and allele G forward 5′-CCT ATC CTA CTT CCC CC-3′ and reverse 5′-AGC AAC ACT CCT CGT CGC AAC-3′. The cycling was of 1 cycle of 95°C/10 min; 40 cycles of 95°C/60 s, 59°C/60 s, and 72°C/60 s, 1 cycle of 72°C/7 min. For each reaction, 10 *μ*L mixture comprised 2 *μ*L of each primer (10 pmol/*μ*L), 1x GoTaq Green Master Mix (2X)-Promega (GoTaq DNA Polymerase is supplied in 2X Green GoTaq reaction buffer (pH 8.5), 400 *μ*M dATP, 400 *μ*M dGTP, 400 *μ*M dCTP, 400 *μ*M dTTP, and 3 mM MgCl_2_), and 1 *μ*L of dimethyl sulfoxide (DMSO). The PCR products (139 bp) were analyzed on agarose gel 2% with ethidium bromide (0.4 mg/mL) in electrophoresis.

### 2.3. Statistical Analysis

 The associations between the categorical variables were performed using *χ* ^2^ test. Survival rates were estimated using the Kaplan-Meier method, and survival curves were compared using the log-rank test. The overall survival analysis was performed using the patient's follow-up data, according to the statistical models in conjunction with the Kaplan-Meier Log Rank (Mantel Cox) to assess the death risk in 10.5 years (126 months), and the survival time was calculated as the time from diagnosis to date of last followup for patients who were still alive. All results with *P* < 0.05 were indicative of statistical significance. For the analysis, *BioEstat 5.0* and *GraphPad Prism 5.0* statistical programs were used.

## 3. Results

 The ratio found in the genotypes for the G308A (TNF*α*) polymorphism was 1(AA) : 5(AG) : 2.7(GG), and the genotype frequencies were not in *Hardy-Weinberg* equilibrium (*P* = 0.0351; A allele proportion = 0.41 and G allele proportion = 0.59). The ratio of the genotypes for G1082A (IL10) polymorphism was 1(AA) : 1.9(AG) : 1.08(GG), and the genotypic frequencies were in *Hardy-Weinberg* equilibrium (*P* = 0.7725; A allele proportion = 0.49 and G allele proportion = 0.51) (Table 1). 

 The median follow-up duration in all patients was 37 months; our attention was also calledin to the death/living relationship in TNF*α* AA (0.71) and IL10 AA (1.0) genotypes. A higher percentage of the IL10AA genotype was found in patients with ALL, and they also showed a high ratio of death/live 1.0 (Table 2).

Table 3 shows the survival rate, stratified at different time intervals (25, 50, 75, 100, and 126 months), when compared to allelic frequencies. The TNF*α* gene showed a small difference between their rates, and the TNFA allele had a greater rate in the intervals analyzed.

On the other hand, the IL10 gene showed a greater range wider than the intervals between the rate of their alleles, and the IL10A allele had a better rate survival in the intervals. 

 After analyzing the follow-up results, we found that up to 75 months, the death estimate decreased, showing a higher percentage up to 25 months (18.18%) for IL10A allele, while the IL10G allele had a better life expectancy in all ranges of months analyzed. Regarding the TNF*α* gene, the TNF A allele had the best life expectancy up to 75 months and TNFG allele demonstrated a higher death risk until 50 months (Table 3).

**Figure 1:**
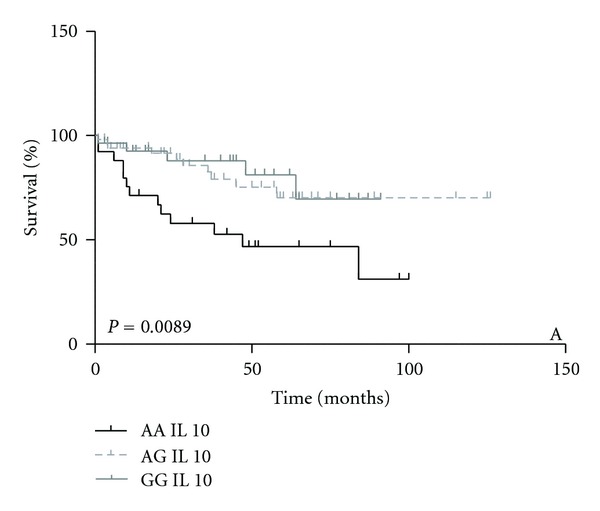
Survival analysis in ALL patients stratified by *IL10* (AA/GA/GG) genotypes.

 Patients with IL10AA genotype showed a worse survival compared with the others (*P* = 0.0089), indicating a survival rate of 44% up to 100 months, while the IL10AG and IL10GG genotypes had survival rate of 69% up to 126 months (Figure 1).

 Patients with TNFAA genotype showed a shorter median survival time (60 months) compared with the others genotypes (TNFAG and TNFGG) (graph not shown).

**Figure 2:**
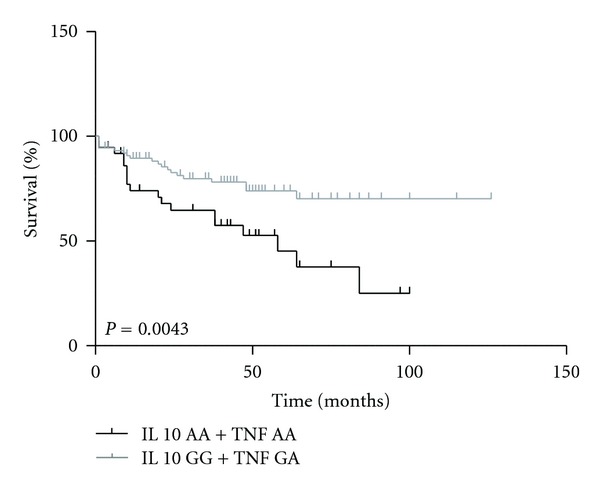
Survival analysis in ALL patients stratified by *IL10* (AA) + *TNF*α** (AA) and *IL10* (GG) + *TNF*α** (GA) genotypes.

 The patients who had IL10AA + TNFA genotypes demonstrated a lower survival rate when compared with IL10GG + TNFAG genotypes (*P* = 0.0043). After the 50th month, the survival percentage for the patients with IL10AA + TNFAA genotypes was 38%, while it was 75% for the IL10GG + TNFAG genotypes (Figure 2).

## 4. Discussion

 Nowadays, fewer studies propose to study polymorphisms in TNF*α* and IL10 genes in relation to prednisone glucocorticoid in acute leukemia during childhood. This is regrettable once the prednisone is the main drug given during the induction phase in leukemia. This study was the second in challenging the -1082 (IL10 gene) and -308 (TNF alpha gene) polymorphisms, together, in leukemia. The death cause in some patients was due to the patient's reaction in the development of leukemia, acquiring infections due to individual conditions as well as the nutritional state before the treatment and also accentuated by the toxicity to methotrexate (MTX).

 Regarding the G1082A (IL10) polymorphism, Edwards-Smith et al. [17] showed that patients homozygous for the IL10G allele showed high plasma levels of IL10. This finding is also suggested in the study of Lauten et al. [18] that evaluated 135 patients, mostly adults, with ALL in accordance with protocols ALL-BFM 86 and BFM-90 in multicenter trials, according to their response to prednisone (based in Schrappe et al. 2000 [19]) and with stratification into risk groups, which was made, predominantly, according to the leukaemic cell mass estimate (the so-called risk factor, the composite variable calculated from the initial blast count in the peripheral blood, and the sizes of liver and spleen below the costal margin) and the initial treatment response, event-free survival (EFS), and relapse risk. In our study (based on GBTLI99 protocol) there is no definition for response to prednisone, because there has been no study for this purpose. However, no study (based on GBTLI99 protocol—initial dose of prednisone 40 mg/m^2^/day) established a background of prednisone response.

 Lauten et al. (2002) [18] found that patients showing poor response were stratified into the high-risk group and therefore underwent a more intensive treatment, suggesting that patients with IL10GG may have a lower risk when there is a poor response to prednisone. 

 In terms of overall survival, we found in this study that older patients (>15 years) showed worse survival rates, although it has not been statistically significant and that the IL10AA genotype had worse survival under protocol GBTLI99. 

 In this study, the TNFGA genotype was present in a higher ratio among patients with the CD10+ (calla) marker (*P* = 0.0296). Once the CD10+ marker is a variable of good prognosis and the TNF GA genotype presented best survival, we suggest that these patients with these markers (CD10+ and TNF GA) have a better performance in the treatment of ALL.

 The monitoring of survival, through the divisions of the months, showed that both alleles IL10A and TNF G had the highest rate for survival during the period of 126 months, and that the IL10A allele, up to 25 months of followup, showed the highest percentage of risk estimate (18.18%) in the occurrence of deaths. 

 On the other hand, when the G1082A and G308A polymorphisms were correlated with respect to survival, the patients with both genotypes TNFGA + IL10GG showed high overall survival, as opposed to Lauten et al. [18], which found no statistical significance for this comparison.

 In other related studies [20, 21], we found no associations with the G308A (TNF*α*) polymorphism, perhaps due to the TNF expression that can be modulated by several interleukins (not evaluated in this study). However, we suggest that the TNF A allele be involved in worse survival in ALL during childhood. 

 We also suggest that patients presenting IL10AA and TNFAA genotypes have high risk that could lead to poor survival. The results showed no association for TNF*α* gene, but the TNFAA genotype indicated a high rate of death (0.71; Table 1) and worse overall survival. 

 However, until today, few studies have found an association between the rare TNFAA (-308) genotype involved in an increased production of TNF protein and the worse survival. 

Bel Hadj Jrad et al. [13] evaluated 194 patients with non-Hodgkin's lymphomas (NHL) and 160 controls, associating a risk increase in the development of NHL for the TNFAA genotype. The TNFAA genotype can be related to low survival of patients with ALL due to the adenine position (-308) at the TNF gene, and a correlation with a higher protein expression of TNF [22, 23] has been shown. An increased TNF level could impair the efficiency of the antitumor cellular immune response, and TNF can induce the apoptosis by natural killer cells [24].

 The knowledge of these polymorphisms in the development of leukemic diseases could optimize, in the future, the innovative therapies using miRs to silence genes according to the worst response to prednisone and enhance studies such as that of Zhang et al. [25] who analyzed the expression of different genes, using eight miRs for the stratification of pediatric patients with ALL in relation to relapse risk.

 The knowledge of the pathogenesis and survival monitoring of patients with ALL is still unclear. It is necessary to analyze other studies, using other chemotherapeutic protocols to approach this issue.

## Figures and Tables

**Table 1:** Patient characteristics with TNF (G308A) and IL10 (G1082A) polymorphisms in ALL childhood (*n* = 105).

	TNF*α* AA *n* (%)	TNF*α* AG *n* (%)	TNF*α* GG *n* (%)	IL10 AA *n* (%)	IL10 AG *n* (%)	IL10 GG *n* (%)	*P*
Gender	12 (11)	61 (58)	32 (31)	26 (25)	51 (48)	28 (27)	
Male	5 (4.7)	25 (23.9)	12 (11.4)	11 (10.5)	22 (21.0)	11 (10.4)	0.2953
Female	7 (6.7)	36 (34.3)	20 (19.0)	15 (14.3)	29 (27.6)	17 (16.2)	0.1441
Immunophenotyping							
Calla (CD10+)	4 (8)	30 (60.5)	16 (31.5)	14 (28.6)	23 (46.9)	12 (24.5)	**0.0296** ^‡^

*P*  value by *χ* ^2^ test, ^‡^ *P* value significant, and Calla (CD10+) in 49 patients.

**Table 2:** Patient characteristics according tothe monitoring statusin ALL (*n* = 105).

	TNF*α* AA *n* (%)	TNF*α* A *n* (%)	TNF*α* GG *n* (%)	IL10 AA *n* (%)	IL10 AG *n* (%)	IL10 GG *n* (%)	*P*
Followup							
<37 months	5 (4.7)	31 (29.5)	16 (15.3)	15 (14.3)	27 (25.7)	10 (9.5)	**0.0358** ^‡^
≥37 months	7 (6.7)	30 (28.5)	16 (15.3)	11 (10.5)	24 (22.8)	18 (17.2)	0.4332
Living	7 (6.7)	46 (43.8)	24 (22.9)	13 (12.4)	41 (39.0)	23 (22.0)	0.3484
Death	5 (4.7)	15 (14.3)	8 (7.6)	13 (12.4)	10 (9.5)	5 (4.7)	0.0725
Ratio (D/L)	0.71	0.32	0.33	1.0	0.24	0.21	

*P* value by *χ* ^2^ test, ^‡^ *P* value significant, L: living, and D: death.

**Table 3:** Monitoring of survival according to percentage of allelic frequencies (*n* = 105).

Followup	TNF*α* A (%)	TNF*α* G (%)	IL10 A (%)	IL10 G (%)	*P*
25 months					
Survival until	84.93	83.87	81.82	91.03	0.6582
Risk estimate	16.07	16.13	18.18	8.97	0.2884
Rate (CI_95%_)	84.93 (76.72–93.14)	83.8 (76.40–91.35)	81.82 (73.20–90.43)	91.03 (84.68–97.37)	
50 months					
Survival until	80.82	76.34	74.03	84.62	0.4630
Risk estimate	4.84	8.97	9.52	7.04	0.3851
Rate (CI_95%_)	80.82 (71.79–89.85)	76.35 (67.71–84.98)	74.03 (64.23–83.82)	84.63 (76.61–9262)	
75 months					
Survival until	78.08	75.27	71.43	81.84	0.5215
Risk estimate	3.39	1.41	3.51	3.28	0.9686
Rate (CI_95%_)	78.08 (68.59–87.57)	75.27 (66.50–84.04)	71.43 (61.34–81.52)	81.84 (73.22–90.46)	
100 months					
Survival until	78.08	75.27	71.43	81.84	0.5215
Risk estimate	0	0	0	0	
Rate (CI_95%_)	78.08 (68.59–87.57)	75.27 (66.50–84.04)	75.27 (61.34–8152)	81.84 (73.22–90.46)	
126 months					
Survival until	78.08	75.27	71.43	81.84	0.5215
Risk estimate	0	0	0	0	
Rate (CI_95%_)	78.08 (68.59–87.57)	75.27 (66.50–84.04)	75.27 (61.34–8152)	81.84 (73.22–90.46)	

Log-rank test: TNF*α*, *P* = 0.7117, and IL10; *P* = 0.1069; CI_95%_: confidence interval in 95%.
